# A Best–Worst Measure of Attitudes toward Buying Seabream and Seabass Products: An Application to the Island of Gran Canaria

**DOI:** 10.3390/foods10010090

**Published:** 2021-01-05

**Authors:** Javier Cantillo, Juan Carlos Martín, Concepción Román

**Affiliations:** Institute of Tourism and Sustainable Economic Development, University of Las Palmas de Gran Canaria, 35017 Las Palmas de Gran Canaria, Spain; jcarlos.martin@ulpgc.es (J.C.M.); concepcion.roman@ulpgc.es (C.R.)

**Keywords:** best worst scaling, satisfaction, importance, attitudes, seabream and seabass products, island of Gran Canaria

## Abstract

Attitudes are important key drivers that affect consumers’ seafood consumption. The present investigation used a best–worst scaling approach to measure the level of importance and satisfaction of consumers’ attitudes towards the purchase of seabream and seabass in Gran Canaria (Spain). The investigation also compared the results of the best–worst scaling (BWS) approach with those of the traditional Likert-scale method and offers a different perspective of the results using an Importance–Satisfaction Analysis (ISA). The results indicate that the most important attributes concerned the hygiene and safety of the product, the health benefits, the freshness, the taste and the nutrients. At the same time, these attributes were ranked as those which satisfied consumers the most. However, some of the results obtained from the methodologies differed. The results suggest that, in the Likert-scale task, respondents might be overstating the importance and satisfaction of the attributes; while in the BWS, consumers were forced to evaluate a trade-off in the selection of the best and worst attributes in each scenario, so the task impeded, in principle, to define every attribute as very important and providing a high satisfaction. As a result, we consider that BWS offers more reliable and clearer results than traditional Likert-scale experiments.

## 1. Introduction

Fish consumption behaviour depends on determinants such as attitudes towards fish consumption, social norms and perceived behavioural control [1,2,3]. Amongst these, attitude is the main element that explains fish consumption behaviour [4,5,6,7,8,9] and can be defined as a psychological trend expressed by evaluating an individual entity (for example a seafood product) with a certain degree of favour, likeness or satisfaction [10]; whereas social norms refer to the social pressure to perform a particular behaviour [1], and the perceived behavioural control reflects past experiences that facilitate conditions or anticipate difficulties [11].

The attitude depends on the different characteristics of the products such as the sensory qualities (freshness, texture, taste and smell), health benefits and price [2]. The evaluation of these determinants may be either positive or negative and represent respectively drivers or barriers to fish consumption behaviour. Common drivers for fish consumption related to attitudes are: (1) good taste [4,6,12,13,14,15] and (2) freshness (quality), ease of preparation or high nutritional value [6]. On the contrary, the proven major barriers to fish consumption are related to unpleasant sensory qualities of seafood such as distasteful smell, unpleasant taste or texture and the presence of bones [3,4,6,13,16]; although some authors have found that price is the most relevant barrier to seafood consumption [3,6,13,17].

Moreover, in a literature review of 49 studies that assessed consumer behaviour on the purchase of fish and seafood products [18], the authors found that the main drivers for fish consumption were the sensory liking of fish, perceived health benefits and fish-eating habits, while the most important barriers were the sensory disliking of fish, health risk concerns, high price perceptions, lack of convenience, lack of availability of the preferred fish products and lack of knowledge in the selection and preparation of fish.

The European Union (EU) is the largest trader of fishery and aquaculture products (FAPs) in nominal terms [19]. From the countries that constitute the EU, Spain has the third-highest per capita consumption and is the third-largest spender of FAPs, while it also constitutes by far the largest producer in the EU in terms of volume for farmed species [20]. In addition, Spain is the fourth largest country in the world with respect to the total production of seabream and seabass, which are the second and third most important species of Mediterranean aquaculture in terms of production after trout [21].

In particular, the Canary Islands represent the third-largest Spanish region in terms of production of farmed fish species, accounting around 25% and 15% of seabass and seabream produced in Spain, respectively [22]. Despite this, the average fish consumption in the Canary Islands is below the national average [23]. Thus, given the large impact on the Canary Islands on the national production of seabream and seabass species, it is important to understand why this relevance in terms of production is not aligned in terms of consumption. To this end, it is important to analyse consumer preferences and attitudes towards these products, in order to facilitate the implementation of strategies that could increase the consumption of these two relevant species. In addition, a better understanding of the internal market for FAPs enables operators to improve their competitiveness and adopt or modify their current strategies on the basis of consumer demand to strengthen and expand the internal market, thereby encouraging job creation [24].

In this investigation, the main objective was to measure the level of importance and satisfaction of certain consumer attitudes towards the purchase of seabream and seabass in Gran Canaria, the second most populated island in the Canary Islands, with a population of 851,231 in 2019 [25].

Another objective of this investigation was to evaluate alternative approaches to survey response mechanisms that can lead to more robust results. Thus, we compared the results of the traditional widely used Likert-scale responses in which consumers were asked to rate their level of importance and satisfaction for certain attitudes, perceptions and experiences on a scale from 1 to 9; with that obtained from best–worse scaling (BWS) methods, initially proposed by Finn and Louviere [26]. In this case, consumers were presented with different scenarios from which they must select the most and least important attributes as well as those from which they were more and least satisfied with, regarding their last shopping experience of seabream/seabass.

The purpose of this comparative analysis relies on shedding light on some of the disadvantages of the traditional Likert-scales, in which how a respondent evaluates their position on that scale varies across respondents. For example, what is sufficient to make a respondent very satisfied may not be enough to satisfy others [27], which affects the means and the variance of the estimates obtained from these type of surveys [28]. Also, Likert-scale questions allow consumers to take shortcuts in the tasks by rating everything as good or bad while also making it difficult to understand the priority of the various issues assessed [27]. On the other hand, a benefit of the BWS is that respondents are forced to discriminate among items and cannot constantly select the middle or endpoints of the scale [29]. The answers are also less ambiguous, as people are usually clearer about extreme options, and it seems easier for respondents to respond to the questionnaire task compared to other methods [30,31]. In addition, BWS is better at determining the relative impact of a large number of attributes, in particular, qualitative effects [27].

Finally, another objective of the paper was to provide a different perspective of the results in absolute terms of the BWS estimates and Likert-scale ratings, using a similar approach to the common Importance–Performance Analysis (IPA) [32], replacing the performance dimension by the satisfaction dimension, naming this as an Importance–Satisfaction Analysis (ISA). This analysis facilitates the interpretation of the most important attributes by using a two-dimensional plot and makes it easy to understand the comparison between the values for importance and the satisfaction of the different attributes.

The framework suggested in this paper allows the simultaneous calculation of both satisfaction and importance constructs; a process that, if not more difficult, is much harder using other approaches [27]. The BWS methodology has been previously used to evaluate meat consumption habits in households with and without children in Italy, by analysing the relative importance of 12 meat choice purchasing attributes; finding that price and animal welfare were found to be the most relevant attributes [33]. Moreover, other authors used the Importance–Confidence Analysis (ICA) adapted from the Importance–Performance Analysis (IPA) to understand the level of importance and confidence in the production and source attributes of seafood purchases in the US [34]. However, according to our best knowledge, this is the first time that the BWS methodology and Importance–Satisfaction Analysis has been used to analyse attitudes towards fish consumption. Similarly, this investigation constitutes the first study that analyses and ranks attitudes towards the consumption of seabream and seabass in Gran Canaria.

The results provide key insights into the attributes that conform the attitudes that should be highlighted in the production and marketing initiatives for seabream and seabass products in Gran Canaria. In addition, researchers, academics and institutions could also benefit from the results to guide the extent of future research. The rest of the paper is organized as follows: Section 2 describes the materials and methods; Section 3 details the results; Section 4 discusses the results; Section 5 provides some concluding remarks.

## 2. Materials and Methods

The data used in this research were obtained from surveys conducted online using the Google Forms platform. The surveys were administered between 28 April and 14 June of 2020 to adults living in Gran Canaria, Spain, who were responsible for buying food from their homes and who were consumers of seabream and seabass species. The survey was distributed through emails directed to all the population associated with the University of Las Palmas de Gran Canaria, the main public university of the island. A first, an e-mail was sent to introduce and explain the questionnaire in April, and a second e-mail was sent two weeks later to remind the community about the questionnaire. To incentive the participation of the public, in the emails sent, respondents were informed about a prize to be raffled amongst them.

Respondents interested in filling the survey were first asked to confirm that they were residents of the island, that they were responsible for buying food at home and that they were consumers of seabream/seabass. Only those who fit the criteria were allowed to continue with the questionnaire (351 respondents). Next, questions were asked regarding their patterns of consumption and preferences for seafood, fish and seabream and seabass (see Table 1).

Table 1 shows some descriptive statistics on the patterns of consumption of respondents. Results indicate that more than 81.2% consumed seafood at home at least once a week, while more than 52.7% consumed seafood outside the home at least once a month. Specifically, for seabream and seabass, the pattern of consumption was more similar to the consumption of seafood outside the home than for home consumption, with approximately 59.5% consuming seabass or seabream at least once a month. Moreover, regarding the most consumed species, we found that tuna followed by hake, seabream, salmon and seabass were the most popular species consumed on the island. Finally, with regard to the places where respondents bought their fish products, around 86% bought them in supermarkets, while around 55% bought them at markets. A small proportion bought their fish directly from fish companies or fishermen.

After that, a series of traditional ratings-based tasks were presented to understand the importance and satisfaction of different attitudes when buying seabream and seabass species. Respondents were asked first, to determine on a scale from 1 (absolutely not important) to 9 (extremely important) how important 16 different attributes were with respect to their last purchase of seabream/seabass. Similarly, after that, using a similar scale from 1 (completely unsatisfied) to 9 (completely satisfied) they were asked to rate how satisfied they were with their last purchase of seabream/seabass according to each one of the same 16 attributes presented previously. The attributes included were related to health and nutritional issues, safety issues, sustainability issues, sensorial characteristics, convenience characteristics, social behaviour characteristics and the price. These attributes were developed following an extensive review by the authors of the relevant literature. The attributes included were: (1) eating fish is healthy; (2) the product has many nutrients; (3) is easier to digest than red meat; (4) hygiene and food safety of the product; (5) more sustainable than red meat; (6) flavour; (7) knowing that the fish is fresh; (8) easy to prepare; (9) easy to buy; (10) the bones are not a problem; (11) the size (ration) of the seabream/seabass is appropriate; (12) the fishmonger can prepare it as wished; (13) it can be bought 365 days of the year; (14) custom or habit since child; (15) my close family and friends also eat seabream/seabass; (16) price.

**Figure 1:**
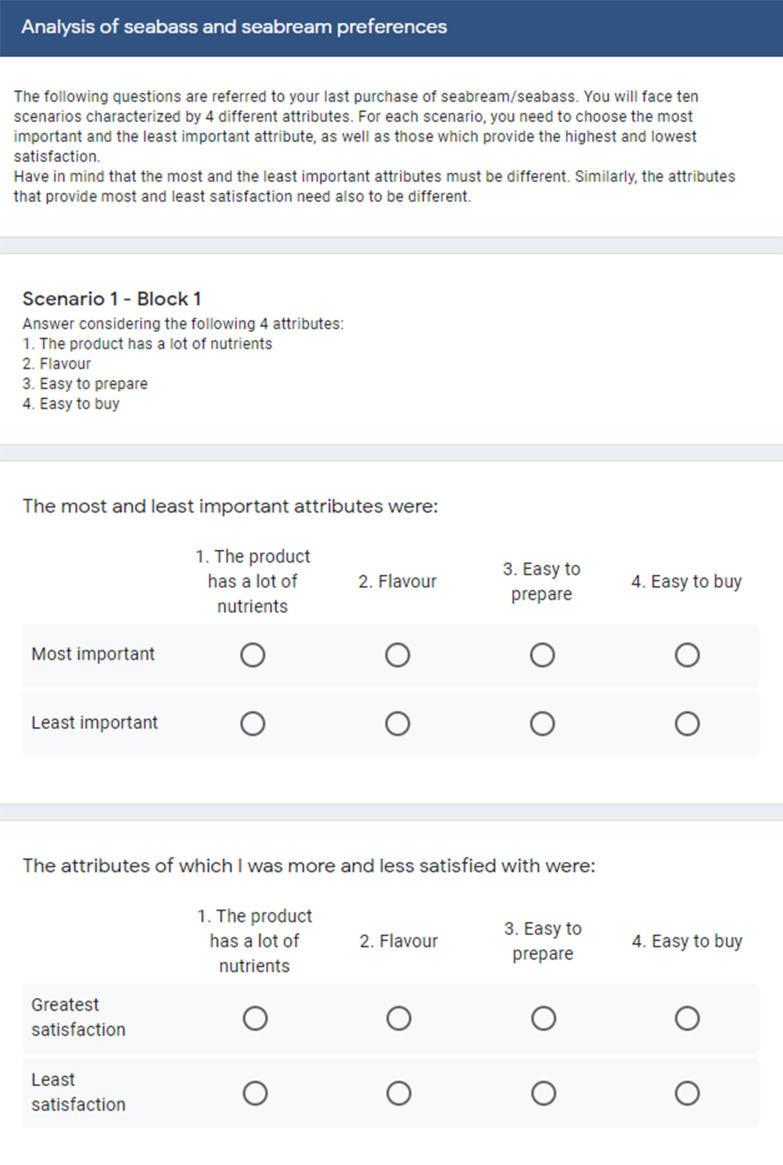
Best–worst measurement of attitudes towards the purchase of seabream and seabass.

After completing the rating tasks, respondents were asked to execute some best–worst case 1 tasks. In total, respondents were asked to make choices in 10 different scenarios, selecting according to their last purchase of seabream/seabass, the most and least important attributes from the alternatives, as well as the attributes from which he/she was most and least satisfied with. Each one of these scenarios (see Figure 1 for an example) consisted of 4 alternatives built out of the same set of 16 attributes that were evaluated for the traditional rating tasks.

A balanced incomplete block design (BIBD) was used to reduce to 10 the tasks required for each respondent. We used the software package “crossdes” and the “findBIB” function in the statistical software R [35], from which we obtained a set of 20 choice tasks for the 16 attributes considered, which were divided into two blocks of 10 tasks each, in order to facilitate the survey administration (see the Appendix A for the full list of tasks per block). Finally, 167 respondents answered to the tasks of Block 1 and 184 to those of Block 2. Our design ensured that: (1) every attribute was shown at least once per block, (2) every selection task included four attributes, (3) each attribute appeared five times considering all the 20 selection tasks and (4) every attribute co-occurred exactly one time with every other attribute over the selection tasks.

Thus, a total of 351 respondents answered to the BWS experiment and considering that each one of them answered to 10 different scenarios with four different choices for each one (most important, least important, most satisfied, least satisfied), our final sample of pseudo-individuals for the estimation of the models were 14,040.

Moreover, in the questionnaires, respondents also answered questions about the image of aquaculture products, which are not discussed in the present investigation. Finally, the questionnaire ended with a series of questions concerning the socio-demographic features of respondents. Table 2 includes a description of the 351 respondents of the survey. Approximately 47% of respondents were between 18 and 35 years of age, while approximately 41% were older than 46 years of age. The majority of respondents were female (60.7%) and single (47.9%). Interestingly, more than 81% of respondents had at least a minimum degree of university education. In addition, 39.03% were public sector workers and 36.18% were students. The overwhelming majority of respondents reported that they had incomes below the national average (70.4%).

The conceptual framework of the methodologies used are explained below.

### 2.1. Best–Worst Scaling (BWS)

The analysis of BWS response data was based on the random utility theory (RUT) [36,37] that suggests that consumers select the alternative that provides them with the highest utility. This utility consists of two components: a systematic and deterministic measurable component and a random component. While the systematic part depends on the alternatives’ attributes as well as on the individual’s socio-economic characteristics, the random part represents the unobserved attributes.

The BWS was introduced by Finn and Louviere [26] as a method of data collection that can prevent and overcome certain limitations of rating-based methods and similar measurement methods. One of the main disadvantages that rating scale experiments exhibit is that respondents are usually biased to select the middle or endpoints of the scale. Thus, the comparison between items is usually obscured. In addition, it is extremely difficult to understand what rating scales values mean, and rating scales are frequently unknown about their reliability and validity [38].

Louviere [39] proposed three different BWS cases, all of which show differences in the nature and complexity of the items selected. In this case, we used the simplest BWS case (case 1) to determine the relative values for each item in the list. In this case, researchers must first select a list of objects and create choice sets, and then individuals (on a subjective scale) are asked to choose the best and worst options in these sets.

Some psychological problems may arise if the size of the choice sets is not consistent. Balanced incomplete block designs are commonly used to solve this issue. The BIBD is a type of design that ensures that selection sizes are equal, each selection option appears equally often and co-appears equally as frequent as each of the other choice options. This reduces the chance of respondents making incorrect assumptions about objects based on design aspects. More information and a detailed guide of choice set construction in BIBDs can be consulted in Reference [40].

#### Implementation of the BWs

We estimated a multinomial logit model. For each choice set, we considered 8 utility functions: the first 4 utility functions for the importance parameters and the other 4 utilities for the satisfaction parameters. The dataset resembled that of an unlabelled experiment to measure importance and satisfaction in which the alternatives *i* (the subindices in Equations (1) and (2)) simply reflected the specific position for the task (1 = top, 2 = second from the top, 3 = second from the bottom, 4 = bottom). Each task provided two observations for modelling: one for the best choice and the other for the worst choice. The analysis of best choices was based on utility maximisation, whereas the worst choices were based on the maximisation of the negative of the utility. As a result, when coding the explanatory attributes, we considered (−1) instead of (1) for the worst-case observation. Thus, the dummy coded 1 or the negative dummy coded −1 were used when the attribute represented the alternative *i* for the best and worst case choices.

For modelling purposes, the four alternatives shown in the choice task were always available when asking for the best options. However, the best-chosen option was no longer available when choosing the worst option, because the same alternative cannot be evaluated at the same time as the best and the worst option. This means that the availability of alternatives for the worst choices was determined by the previous best choices of the individual and, in our case, there were always three alternatives available for the worst choices. The utility functions *U* for both, importance and satisfaction of the alternative *i* are:

(1) $$U_{i}^{Imp}=\;ASC_{i\;}+\;\overset{15}{\underset{k=1}{\sum }}\beta \;Imp_{k}\times Imp_{ik}\;\mathrm{with}\;i=1,2,3,4$$

(2) $$U_{i}^{Sat}=\;ASC_{i\;}+\;\overset{15}{\underset{k=1}{\sum }}\beta \;Sat_{k}\times Sat_{ik}\;\mathrm{with}\;i=1,2,3,4$$

where the explanatory attributes are defined as: *ACS_i_* = reflect the positional and other ordering effects; *Imp_ik_* = 1 (or −1) if the attribute k is shown in alternative *i* for best (or worst) choices and 0 otherwise; *Sat_ik_* = 1 (or −1) if the attribute *k* is shown in alternative *i* for best (or worst) choices and 0 otherwise.

Since our study included 16 attributes, we have included 15 dummy variables considering that the last one acts as a reference. The coefficients; *βImp_k_* and *βSat_k_* can be understood as the degree of importance and satisfaction for the attribute *k*, respectively. The interpretation of the parameters was based on the reference with effect “0”, which corresponded to the attribute “(16) price”. Therefore, negative parameters meant that the respective attributes were less important or satisfying than the price, while positive parameters indicated that they were more important or satisfying than the price. Similarly, the statistical significance of the parameters indicated differences in the level of importance or satisfaction with respect to attribute 16.

We used four alternative specific constants (ASCi) to reflect the positional and other ordering effects that might exist within the data, being the constant of the alternative presented in the fourth place (ASC4) normalised to 0. Moreover, the observed utility component, the multinomial logit model, included an error term. The free software “Biogeme” was used to estimate the multinomial logit model [41].

### 2.2. Importance–Satisfaction Analysis

**Figure 2:**
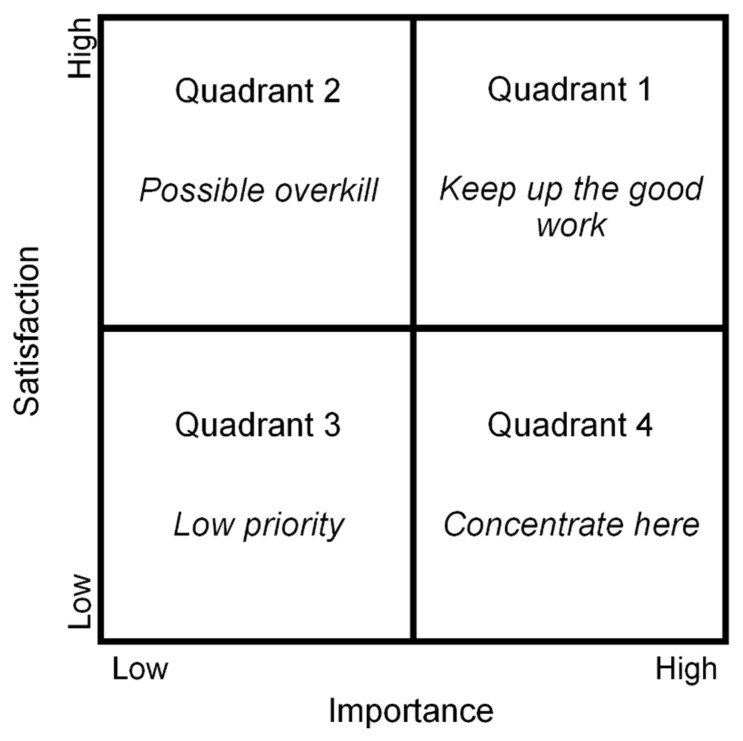
Importance–Satisfaction Analysis (ISA) plot.

In order to better understand the results of Likert-scale and BWS tasks, we used an Importance–Satisfaction Analysis (ISA) in which the main objective is to identify the most important/satisfying attributes using a two-dimensional plot that facilitates the interpretation of the data. Figure 2 shows the ISA plot in which the attributes in the plot are divided into four quadrants.

Each one of the attributes was plotted in a coordinate system according to the means of the ratings given by respondents in the Likert-scale experiment and the values obtained for the parameters in the multinomial logit model estimated for the BWS results. Moreover, the axes for the quadrants can be obtained according to different methods such as scale averages or data averages. In our study, the data average values were used for both the Likert rating scale and the estimated parameters obtained for the BWS method. The chart is known as the data centred quadrant model representation (DCQMR) [32].

The first quadrant, “Keep up the Good Work” includes high satisfaction and importance attributes that indicate that these attributes are performing well. The characteristics of the second quadrant “Possible overkill” were good in terms of satisfaction, but they are not very important. In addition, the “low priority” of Quadrant 3 included attributes that were not very good in terms of satisfaction but were considered relatively insignificant in importance for respondents. Therefore, managers should not be too concerned about these attributes. Finally, Quadrant 4 “Concentrate here” was the most important region in the plot, in which the attributes are considered to be of high importance but were under-performing and, thus, were those in which the focus should be placed [42].

## 3. Results

### 3.1. Rating Scale Results

Regarding the rating tasks, respondents were asked to rate their degree of satisfaction and the importance level of each one of the 16 attributes using a nine-point Likert scale. The mean, median and standard deviation (SD) values for each one of them are presented in Table 3.

We found that three of the 16 attributes had a median of nine out of a maximum of nine for importance ratings with five having a median of eight, six a median of seven and two a median of six. Similar results were found for the satisfaction question, with three attributes having a median of nine, four having a median of eight, eight having a median of seven and only one having a median of six. Moreover, for half of the attributes, the mean importance value was higher than the mean value for the satisfaction questions.

The top three rated attributes for importance were “Hygiene and food safety of the product”, “Eating fish is healthy” and “knowing that the fish is fresh”; while the bottom three rated were “It can be bought the 365 days of the year”, “The bones are not a problem” and “My close family and friends also eat seabream/seabass”. On the other hand, on the basis of the degree of satisfaction, the top and bottom three rated attributes were the same as those judged by importance, except for the inclusion of the attribute “Custom or habit since child” instead of “It can be bought the 365 days of the year” for the bottom three.

### 3.2. Best–Worst Scale Results

Initially, a multinomial logit model based on the best–worst tasks was estimated considering all attributes (see Appendix A for the results). With the results of this initial model, we estimated the 95% confidence intervals for the satisfaction and importance values of the different attributes. Based on these, we estimated a new model (see Table 4) restricting as equal the importance and satisfaction parameters of an attribute whose 95% confidence intervals overlap. Also, in the new model, we fixed as 0 the importance and satisfaction parameters for the attributes that were not significantly different from the base attribute “Price” according to a significance level of 0.1. Table 4 shows at the beginning the attributes in which the importance and satisfaction parameters were fixed as equal, following those in which they differ. 

We found that in seven of the 15 attributes, the importance and satisfaction confidence intervals overlapped, which is why for these cases, both parameters were restricted to be equal. In all cases, they turned out to be statistically significant (with a level of 0.05), indicating that the importance and satisfaction of these attributes were different from the importance and satisfaction of the “Price” attribute. From these attributes, three of them (“The bones are not a problem”, “The size (ration) of the seabream/seabass is appropriate” and “Custom or habit since child”) had a negative sign, suggesting that these attributes were less important and offered less satisfaction than the “Price” attribute; while four had a positive sign (“Eating fish is healthy”, “The product has a lot of nutrients”, “Flavour”, “Knowing that the fish is fresh”), indicating that they were more important and offered higher satisfaction than “Price”.

Moreover, for seven of the 15 attributes, different parameters were estimated for the importance and satisfaction estimates. Specifically, with respect to the importance results, we found that all values were statistically significant (with a level of 0.1), indicating that the importance of these attributes was different from the importance of the “Price” attribute. From them, six attributes had a negative sign and were statistically significant, suggesting that these attributes were less important than the “Price” attribute, while the remaining significant positive attribute “Hygiene and food safety of the product” resulted in being more important than the “Price”. In addition, regarding the satisfaction results, three of the attributes were found to be significant and negative, implying that consumers were less satisfied by these attributes in respect to the “Price” attribute, while the attributes “Hygiene and food safety of the product” and “Easy to prepare” were statistically significant and positive, showing that consumers were more satisfied by them in comparison to the “Price” attribute.

The results indicate that the first three ASCs were statistically significant and positive, which meant that respondents were more likely to select one of the first three items shown in the choice set rather than the fourth alternative in terms of the order, ceteris paribus.

### 3.3. Comparing the Approaches

**Figure 3:**
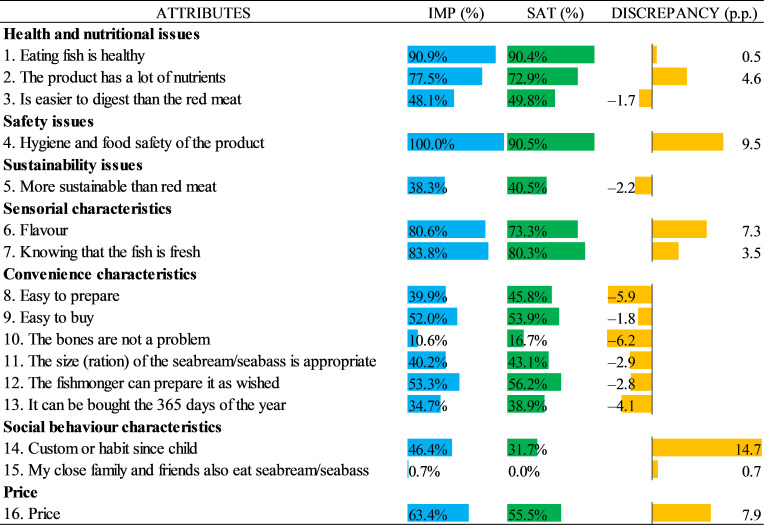
Traditional Likert-scale relative importance and satisfaction results.

**Figure 4:**
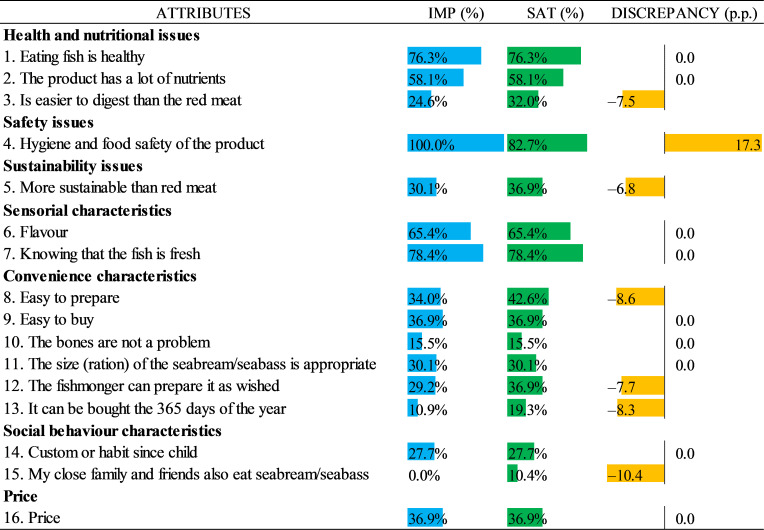
Best–worst relative importance and satisfaction results.

In order to illustrate the results more precisely, we normalised the importance and satisfaction estimates of the results presented in Table 3 and the model presented in Table 4 by giving the lowest score a 0 and the highest score a 1. This was achieved by taking the difference between each item and the minimum value and dividing the result by the range (the difference between the largest and the smallest values). The results can be seen in Figure 3 for the traditional Likert-scale and in Figure 4 for the BWS. The rescaled results are shown in blue for the importance index, while green for the satisfaction index. In addition, the differences between the two indexes for the same attribute indicate a discrepancy between the relative satisfaction and the importance of the attribute.

From the BWS results, it can be observed that the most important attributes in order were “Hygiene and food safety of the product”, “Knowing that the fish is fresh”, “Eating fish is healthy”, “Flavour” and “The product has a lot of nutrients”, while the least important attributes were “My close family and friends also eat seabream/seabass”, “It can be bought 365 days of the year” and “The bones are not a problem”. In addition, the attributes with the highest and lowest satisfaction levels were the ones same listed as the most and least important, respectively. However, the highest positive discrepancy (higher importance and less satisfaction) was observed for the most important and most satisfying attribute: “Hygiene and food safety of the product”.

In addition, we observed some differences when comparing the rescaled data from the BWS and the traditional Likert-scale. For example, in the BWS results, we found that the second most important and the second most satisfying attribute was “Knowing that the fish is fresh”, while for the Likert scale it was “Eating fish is healthy”; however, for the third position, these two attributes switched position accordingly in each case. A similar situation occurred for two of the top three least important attributes: “The bones are not a problem” and “It can be bought 365 days of the year”. Moreover, the differences increase if the magnitudes of the values are considered, in which almost all values related to the importance and satisfaction of the Likert-scale results were higher than the values of the BWS. Further, the attribute “It can be bought 365 days of the year” with 34.7% was rated as three times higher in importance compared to the results of the BWS (10.9%), while similarly for this same attribute, the value on the Likert-scale for the satisfaction (38.9%) doubled the one found with the BWS (19.3%). Another example occurred with the attribute “Is easier to digest than red meat” which was rated almost twice as important in the Likert-scale results (48.1%) in comparison with the BWS results (24.6%).

Another difference between the two types of data can be found in the discrepancy between the importance and satisfaction results, in which the same attributes can have different results. For example, in the Likert-scale results, it was observed that the highest discrepancy was related to the attribute “Custom or habit since child” that showed higher importance and less satisfaction, whereas in the BWS results, there was no discrepancy at all.

**Figure 5:**
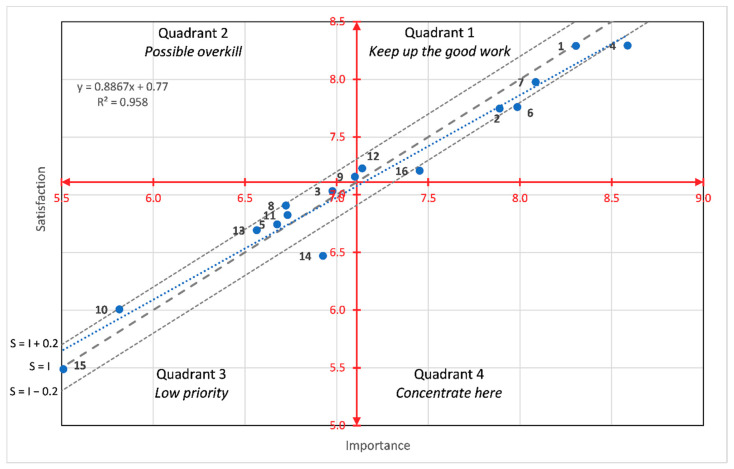
Importance-Satisfaction Analysis based on Likert-scale scores. The red lines represent the axes that divide the quadrants, the blue line the linear adjustment of the points and the grey lines the diagonal lines (S = I, S = I + 0.2, S = I − 0.2).

**Figure 6:**
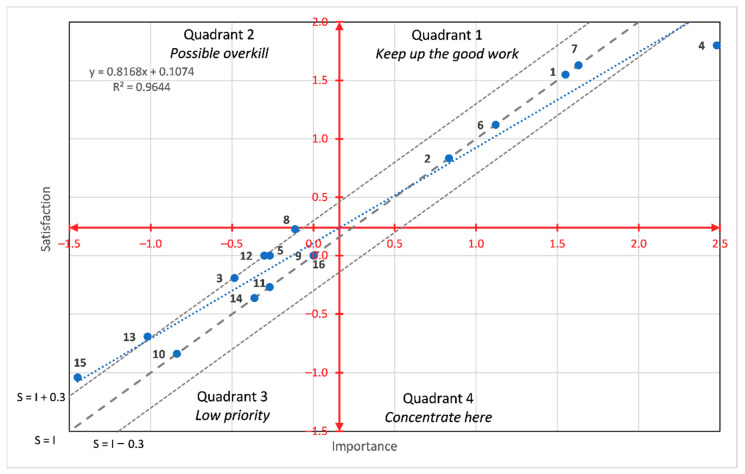
Importance–Satisfaction Analysis based on BWS scores. The red lines represent the axes that divide the quadrants, the blue line the linear adjustment of the points and the grey lines the diagonal lines (S = I, S = I + 0.3, S = I − 0.3).

Moreover, Figure 5 and Figure 6 present a different perspective of the results according to the two-dimensional importance–satisfaction grid. In the figures, the red lines separate the quadrants of the importance–satisfaction analysis according to the average values of the attributes for the Likert rating scale, and the estimated coefficients for the BWS method. In IPA parlance, this is known as the data centred quadrant model representation (DCQMR) [32]. The figures also show three dashed grey diagonal lines that represent the discrepancy analysis [43]. The diagonal line (S = I) is known as the iso-rating line that is characterised because the discrepancy is zero. The line above the diagonal is characterised because the discrepancy is constant and satisfaction is higher than importance, so independently of the quadrant, these attributes are considered as the consumers’ satisfiers. The opposite logic prevails for the diagonal lines below the iso-rating line. Moreover, the figures also include an additional dotted blue line with the regression of the satisfaction values over the importance values. Both figures show that almost all the attributes are located in the quadrants 1 and 3 (except for attribute 9 in the Likert-scale results), indicating either that there is good satisfaction for important attributes or that, if some attributes provide low satisfaction levels, they are not of the highest priority or importance. Additionally, it can be concluded from the figures that importance and satisfaction were highly correlated. The particular case of attribute 9 for the Likert-scale results, which is located in quadrant 2, indicates that it had higher satisfaction than its actual importance.

In general, we observed that apart from the differences in the rankings between the two methods, the magnitude of the importance and satisfaction results of the Likert-scale task were higher than in the BWS task, which suggests that, in the Likert-scale task, respondents might be overstating the importance and satisfaction of the items. This result is related to the socially desirable responding and acquiescence bias [44,45]. Meanwhile, in the BWS, consumers were forced to evaluate a trade-off in the selection of the best and worst alternatives in each scenario, so the task impeded in principle to define every item as very important and very satisfying attribute. As a result, it can be concluded that BWS offers more reliable and clearer results than traditional IPAs based on semantic scales.

In addition, the BWS method forces respondents to consider relative levels of importance and satisfaction, which involves a more direct evaluation between the attributes differentiation than simply rating the attribute’s importance and satisfaction. Thus, the BWS proposed in the paper is very different from the traditional ratings; for example, it can be seen that in the BWS results (Figure 4) there is only one positive discrepancy against six negative discrepancies, while in the Likert-scales results (Figure 3), there is an equal number of positive and negative discrepancies (8). Thus, it can be concluded that the discrimination enforced by the BWS method and the estimates obtained from the model overcame a number of issues that have been cited when researchers use the Likert rating scales [46,47].

## 4. Discussion

In this section, the results of the BWS methodology are considered to propose some marketing implications. The first actions to be considered by the authorities and stakeholders are those related to attributes that have been ranked as the most important, but which level of satisfaction does not have the expected results. The most important attributes concerned the hygiene and safety of the product, the healthiness, the freshness, the flavour and the nutrients that it possesses. At the same time, these attributes were ranked as those from which consumers were the most satisfied. In all cases, however, the level of satisfaction was not at the same level as the importance. The results of the BWS show a relatively lower level of satisfaction with the “Hygiene and food safety of the product” attribute which was ranked as the most important attribute. A study found that in India, the freshness and cleanliness of food products were the most important attributes for food choice and, therefore, suggested that food retailers should focus on satisfying this item [48]. Moreover, a study found in China showed that consumers were most willing to pay for enhanced food safety when purchasing shrimp and imported tilapia [49]. Based on these findings, strategic plans to improve customer satisfaction with the hygiene and safety of these products are very important, especially given that other studies have shown that consumers are willing to pay premiums for safety claims that enhance some aspect of product safety [50,51]. For this purpose, the seabream and seabass industry can follow the example of the salmon industry, where stakeholders have adopted new safety procedures during different phases of production, processing, distribution and wholesale and retail sales in order to meet the growing demand for safe farmed Atlantic salmon [52]. Also, a study found that, for safety reasons, consumers agreed to the idea of using traceability methods and quality control systems in the salmon industry, despite the increase in the cost of the product [53]. This is consistent with another study that found that those with a higher frequency of consumption tend to regard the safety of the product as more important than the price; therefore, the provision of promotional activities underlining the safety of fish can make a significant contribution to increasing fish consumption [54].

The ISA plots for the BWS results indicate that the attributes are already either important and with a high level of satisfaction (Quadrant 1) or with a low level of satisfaction and a low level of importance (Quadrant 3). Nevertheless, although the attributes of the “keep up the good work” quadrant indicate good levels of both importance and satisfaction, it is useful to continually improve them because they can be seen in some circumstances as the main attributes to provide competitive advantages. In the case of the BWS results, the following attributes were located in this quadrant: (1) eating fish is healthy, (2) the product has a lot of nutrients, (4) hygiene and food safety of the product, (6) flavour and (7) knowing that the fish is fresh.

Given the previous results, first, it is important to implement strategies that increase the satisfaction for the health benefits offered by these products. Fish and seafood products are generally perceived as healthy due to the number of their health and nutritional benefits, especially their high content in omega-3 fatty acids and protein as well as their low-fat content [13,55,56,57,58,59]. Nevertheless, consumers also weigh different risks, which could also constitute an obstacle to their consumption [56]. In fact, consumers may simultaneously perceive both the health benefits and the health risks of fish consumption, with an anticipated antagonistic impact on their choices [18]. While health benefits influence positively fish consumption behaviour in terms of their nutritional values and lower risk of diseases, health risks related to chemical contaminants such as mercury have been identified as barriers for fish consumption [2]. Given this, marketing campaigns should focus on increasing the health benefits of fish consumption, as well as explaining how to avoid the possible risks related to their consumption. In addition, several investigations have shown that consumers are willing to pay extras for products highlighting benefits such as the improvement of heart function [60,61] and brain function [60]; therefore, producers should focus on producing fish that contribute to an enhanced health condition in an attempt to increase both their revenue and the perceived satisfaction of costumers for the health benefits associated to fish consumption.

Regarding our finding of the importance given to nutrients, another study also found that the high nutritional value of fish products is an important driver of its consumption [6]. Some of the relevant nutrients found in fish are digestible proteins, vitamins A and D3, trace minerals such as iodine and selenium and n-3 long-chain polyunsaturated fatty acids [62]. Given that some studies have found that consumers are willing to pay extras for products with a high content in omega-3 fatty acids [50,60,63,64] or fortified with beneficial and healthy compounds [62], producers and sellers are encouraged to invest on these types of products.

As far as flavour is concerned, similar results on the importance of this attribute have been obtained in the literature. On one hand, a study on Hawaiian consumers considered that taste was the most important reason to consume seafood and to prefer wild products to aquaculture production [65]. Similarly, another study found that flavour was the second most important attribute for the consumption of farmed seabream [62], while another study argued that taste was one of the most important drivers for eating fish [3]. Given the importance and the fact that satisfaction is not at the same level, some strategies need to be considered in order to improve the taste of the products, such as marketing campaigns highlighting different recipes to cook seafood, which might be more pleasant in terms of flavour than the usual ways of cooking fish.

The freshness of the product is important as sometimes it is associated with its quality [6]. In the literature, several studies have shown a general preference and greater willingness to pay for fresh products over other types of presentations [65,66,67,68,69,70]. A study found that freshness was the most important attribute for farmed seabream consumption in Portugal [62], while another study in Europe found that one of the most important reasons for buying local and European products was its greater freshness [71]. This preference for fresh products implies that efforts should be made to optimize the supply chain for fisheries and aquaculture to ensure that more fresh products are marketed [72]. However, not knowing how to evaluate if the fish is fresh or not can be a barrier to its consumption [56], which is why marketing campaigns should provide a guide for consumers in the assessment of freshness of products.

Finally, although managers should not pay much attention to the attributes located in the “low priority” quadrant, because they are not important or satisfactory, they remain a matter of concern that the authorities should address in the case that some changes are observed.

## 5. Conclusions

The results of the present investigation are a source of valuable information to be used for product improvement and marketing by the various stakeholders involved in the production of seabream and seabass in Gran Canaria. The findings not only help to understand the attributes that consumers consider to be the most important and the level of satisfaction they have with them but also help to understand how the two of them interact together, allowing to determine which attributes should be of greater concern for improving the quality of seabream and seabass products in Gran Canaria.

Comparing the results of the two experiments, in general, we find that the magnitude of the importance and satisfaction results in the Likert-scale task were higher than in the BWS task, which suggests that in the Likert-scale tasks respondents might be overrating the importance and satisfaction of the items; while in the BWS, consumers were forced to select the best and worst alternatives in each scenario. Thus, the task impeded in principle to define every item as very important and very satisfying, and as a result, we concluded that BWS offers more reliable and clearer results.

The results of the analysis indicated that the most important attributes, and those that consumers are more satisfied with, are related to the hygiene and safety of the product, the health issues, the freshness, the flavour and the nutrients it contains. However, in some cases the level of satisfaction assigned to them differed from the level of importance, indicating that actions are needed to improve efficiently the quality of the products, especially for the case of the attribute related to the hygiene and food safety of the product, which was considered to be the most important, but whose level of satisfaction was relatively lower in magnitude, according to the BWS results. In addition, the results of the Importance–Satisfaction analysis for the BWS experiment show that all attributes were either considered as important and have a high level of satisfaction, or low satisfaction and low level of importance, which did not indicate critical issues that should be addressed with higher priority.

The main limitation of the study relates to data collection, as it was collected online by sending invitations to students and staff members related to the University of Las Palmas de Gran Canaria, and although it was clarified in e-mails that the survey could be shared with others outside the university context, most of the respondents were probably somehow related to the university. This also explains why the sample presents a high volume of respondents with a university degree as well as a high number of students. In addition, we used a convenience sampling method because it was not possible to know the population of adults in Gran Canaria that buy seabass and seabream products and that are also responsible for buying the products at their home, which were two mandatory conditions for answering the survey.

One major consideration for future research is to circumvent the main limitation of the study extending the sample to more population segments in Gran Canaria, as well as more regions in the EU and the world. Moreover, future research should establish different types of analysis for farmed and wild products, as attributes, such as safety, might be valued differently by consumers. This was already identified in a study, in which respondents agreed that farmed fish were safer due to the major controls and balanced feeding [73]. Future research should also assess the reliability of the results of the relationship between importance and satisfaction for the different attributes, as attributes may be of little importance for respondents once they have exceeded a particular level of satisfaction. An example of this occurs in the automobile market of some countries, where safety has become less important, as all vehicles sold must comply with minimum safety standards, so they are considered to be safe for this matter [27]. A similar situation might be happening with some of the attributes listed in this study. In addition, the current study also provides interesting issues for authorities, marketers and producers related to farmed seabream and seabass, as they can evaluate the degree of satisfaction that different attributes provide to consumers in relation with the importance that consumers give to these attributes. In a more industrial setting, our study could be extended to analyse specific product formats, selling establishments or even consumers’ characteristics that could determine market segmentation. With enough and adequate data, the model could be enriched with new covariates that provide better insights to the stakeholders. This is a promising area for future research. In any case, the interest of using an alternative approach for the assessment of consumers’ attitudes towards purchase of seabream and seabass will continue. The study is somewhat localised and dealing with an insular population (i.e., islanders), but inferences can be made to other similar geographies in which seabream and seabass consumption is also common.

## Figures and Tables

**Table 1:** Consumption descriptive statistics.

Frequency of Consumption			
Frequency	Seafood and Fish		Seabream and Seabass
	At Home	Outside the Home	
Never/Almost never	1.1%	15.1%	9.4%
Sometimes in a year	1.4%	32.2%	31.1%
Once a month	4.3%	22.8%	23.7%
2 or 3 times a month	12.0%	17.4%	21.7%
Once a week	43.3%	10.5%	11.1%
2 or 3 times a week	37.0%	2.0%	3.1%
Everyday	0.9%	0.0%	0.0%
**Top Three Species Consumed**			
Species	Percentage		
Tuna	19.7%		
Hake	15.0%		
Seabream	12.9%		
Salmon	11.8%		
Seabass	8.6%		
Sole	7.2%		
Cod	5.8%		
Mackerel	4.8%		
Wreckfish	4.4%		
Sama	4.1%		
Other	5.8%		
**Locations to Buy Fish and Seafood (Several Options Possible)**			
Location	Percentage		
Markets	55.0%		
Supermarkets	86.0%		
Fish companies	1.1%		
Fishers directly	5.1%		

Number of respondents: 351.

**Table 2:** Sample descriptive statistics according to age, gender, marital status, education level, occupation and income.

Age Range		Maximum Education Level Reached	
18–25	28.8%	Primary school	1.4%
26–35	18.5%	High school	10.5%
36–45	11.4%	Technician degree	6.6%
46–55	23.9%	University degree	43.3%
56 or older	17.4%	University postgrad	38.2%
**Gender**		**Occupation**	
Male	39.3%	Independent worker	6.0%
Female	60.7%	Public employee	39.0%
**Marital Status**		Private sector employee	14.3%
Single	47.9%	Student	36.2%
Married	34.8%	Unemployed	2.0%
Living with a partner	16.8%	Retired	0.9%
Widow	0.6%	Housekeeper	1.7%
**Income**			
Below national average	13.7%		
Around national average	70.4%		
Above national average	16.0%		

Number of respondents: 351.

**Table 3:** Importance and satisfaction rating task results (Mean, Median and Standard deviation of the attributes).

Attributes	Importance			Satisfaction		
	Mean	Median	SD	Mean	Median	SD
**Health and nutritional issues**						
(1) Eating fish is healthy	8.30	9	1.13	8.29	9	1.19
(2) The product has many nutrients	7.89	8	1.33	7.75	8	1.52
(3) Is easier to digest than the red meat	6.98	8	2.28	7.03	8	2.27
**Safety issues**						
(4) Hygiene and food safety of the product	8.59	9	1.02	8.29	9	1.27
**Sustainability issues**						
(5) More sustainable than red meat	6.68	7	2.18	6.74	7	2.17
**Sensorial characteristics**						
(6) Flavour	7.99	8	1.30	7.76	8	1.46
(7) Knowing that the fish is fresh	8.09	9	1.39	7.98	9	1.52
**Convenience characteristics**						
(8) Easy to prepare	6.72	7	1.83	6.91	7	1.89
(9) Easy to buy	7.10	7	1.69	7.16	7	1.78
(10) The bones are not a problem	5.81	6	2.63	6.01	7	2.56
(11) The size (ration) of the seabream/seabass is appropriate	6.73	7	1.78	6.82	7	1.79
(12) The fishmonger can prepare it as wished	7.14	8	1.93	7.23	8	1.88
(13) It can be bought 365 days of the year	6.56	7	2.18	6.69	7	2.14
**Social behaviour characteristics**						
(14) Custom or habit since child	6.93	7	2.06	6.47	7	2.38
(15) My close family and friends also eat seabream/seabass	5.51	6	2.28	5.49	6	2.33
**Price**						
(16) Price	7.45	8	1.60	7.21	7	1.80

**Table 4:** Best–worst task estimates—Model 2.

**Attributes in Which the Importance and Satisfaction (IMP–SAT) are the Same**						
Attributes	IMP–SAT		t-Stat		*p*-Value	
**Health and nutritional issues**						
(1) Eating fish is healthy	**1.55**		27.36		0.00	
(2) The product has many nutrients	**0.833**		16.63		0.00	
**Sensorial characteristics**						
(6) Flavour	**1.12**		22.03		0.00	
(7) Knowing that the fish is fresh	**1.63**		30.14		0.00	
**Convenience characteristics**						
(10) The bones are not a problem	**−0.839**		−16.9		0.00	
(11) The size (ration) of the seabream/seabass is appropriate	**−0.269**		−5.52		0.00	
**Social behaviour characteristics**						
(14) Custom or habit since child	**−0.363**		−7.43		0.00	
**Attributes in Which the Importance (IMP) and Satisfaction (SAT) Differ**						
Attributes	IMP	t-stat	*p*-value	SAT	I-stat	*p*-value
**Health and nutritional issues**						
(3) Is easier to digest than the red meat	**−0.485**	−7.02	0.00	**−0.191**	−2.89	0.00
**Safety issues**						
(4) Hygiene and food safety of the product	**2.48**	30.39	0.00	**1.8**	25.31	0.00
**Sustainability issues**						
(5) More sustainable than red meat	**−0.268**	−3.96	0.00	0.00	-fixed-	-fixed-
**Convenience characteristics**						
(8) Easy to prepare	***−0.113***	−1.73	0.08	**0.226**	3.59	0.00
(9) Easy to buy	0.00	-fixed-	-fixed-	0.00	-fixed-	-fixed-
(12) The fishmonger can prepare it as wished	**−0.302**	−4.25	0.00	0.00	-fixed-	-fixed-
(13) It can be bought 365 days of the year	**−1.02**	−14.46	0.00	**−0.693**	−10.46	0.00
(15) My close family and friends also eat seabream/seabass	**−1.45**	−19.94	0.00	**−1.04**	−15.59	0.00
**Price**						
(16) Price	0.00	-fixed-	-fixed-	0.00	-fixed-	-fixed-
*Alternative specific constants (ASCs)*	Value		t-stat		*p*-value	
ASC1	**0.178**		5.64		0.00	
ASC2	**0.131**		4.38		0.00	
ASC3	**0.161**		5.62		0.00	
ASC4	0.00		-fixed-		-fixed-	
*Goodness of fit*						
*McFadden’s pseudo R* ^2^ *(ρ* ^2^ *)*	0.194					
*Adjusted McFadden’s pseudo R^2^ (Adjusted ρ^2^)*	0.193					
Final Log-likelihood	−14,053.504					
Number of observations	14,040					

Values in bold font indicate a significance level of 0.05, while those in italics indicate a significance level of 0.1.

## Data Availability

The data presented in this study are available on request from the corresponding author.

## Appendix A

Table A1 shows all the scenarios included in the survey for the Best-Worst experiment.

**Table A1:** Distribution of alternatives in scenarios per block.

**Block 1**				
Scenario	Alternative 1	Alternative 2	Alternative 3	Alternative 4
1	Attribute 2	Attribute 6	Attribute 8	Attribute 9
2	Attribute 1	Attribute 6	Attribute 7	Attribute 16
3	Attribute 1	Attribute 2	Attribute 10	Attribute 12
4	Attribute 2	Attribute 15	Attribute 11	Attribute 5
5	Attribute 4	Attribute 7	Attribute 10	Attribute 13
6	Attribute 4	Attribute 8	Attribute 16	Attribute 12
7	Attribute 14	Attribute 16	Attribute 10	Attribute 11
8	Attribute 14	Attribute 9	Attribute 7	Attribute 3
9	Attribute 6	Attribute 11	Attribute 3	Attribute 12
10	Attribute 1	Attribute 15	Attribute 3	Attribute 13
**Block 2**				
1	Attribute 15	Attribute 8	Attribute 9	Attribute 10
2	Attribute 15	Attribute 7	Attribute 5	Attribute 12
3	Attribute 1	Attribute 14	Attribute 8	Attribute 5
4	Attribute 4	Attribute 6	Attribute 14	Attribute 15
5	Attribute 8	Attribute 7	Attribute 11	Attribute 13
6	Attribute 1	Attribute 4	Attribute 9	Attribute 11
7	Attribute 4	Attribute 2	Attribute 16	Attribute 3
8	Attribute 6	Attribute 10	Attribute 5	Attribute 3
9	Attribute 2	Attribute 14	Attribute 12	Attribute 13
10	Attribute 9	Attribute 16	Attribute 5	Attribute 13

Attributes: (1) Eating fish is healthy; (2) The product has a lot of nutrients; (3) Is easier to digest than the red meat; (4) Hygiene and food safety of the product; (5) More sustainable than red meat; (6) Flavour; (7) Knowing that the fish is fresh; (8) Easy to prepare; (9) Easy to buy; (10) The bones are not a problem; (11) The size (ration) of the seabream/seabass is appropriate; (12) The fishmonger can prepare it as wished; (13) It can be bought the 365 days of the year; (14) Custom or habit since child; (15) My close family and friends also eat seabream/seabass; (16) Price.

Table A2 shows the results of the initial estimated multinomial logit model based on the best–worst tasks. It includes the importance and satisfaction results for the attributes, with their respective t-statistics and *p*-values.

**Table A2:** Best-worst task estimates—Model 1.

Attributes	IMP	t-Stat	*p*-Value	SAT	t-Stat	*p*-Value
**Health and nutritional issues**						
(1) Eating fish is healthy	**1.54**	16.31	0.00	**1.50**	16.82	0.00
(2) The product has a lot of nutrients	**0.860**	9.6	0.00	**0.742**	8.8	0.00
(3) Is easier to digest than the red meat	**−0.553**	−6.5	0.00	**−0.202**	−2.42	0.02
**Safety issues**						
(4) Hygiene and food safety of the product	**2.46**	25.17	0.00	**1.78**	20.38	0.00
**Sustainability issues**						
(5) More sustainable than red meat	**−0.337**	−3.91	0.00	***−0.155***	−1.86	0.06
**Sensorial characteristics**						
(6) Flavour	**1.06**	12.25	0.00	**1.10**	12.99	0.00
(7) Knowing that the fish is fresh	**1.66**	17.8	0.00	**1.53**	17.44	0.00
**Convenience characteristics**						
(8) Easy to prepare	**−0.182**	−2.11	0.03	**0.222**	2.67	0.01
(9) Easy to buy	−0.0997	−1.18	0.24	0.100	1.20	0.23
(10) The bones are not a problem	**−0.982**	−11.39	0.00	**−0.778**	−9.23	0.00
(11) The size (ration) of the seabream/seabass is appropriate	**−0.413**	−4.79	0.00	**−0.199**	−2.35	0.02
(12) The fishmonger can prepare it as wished	**−0.367**	−4.14	0.00	−0.0310	−0.36	0.72
(13) It can be bought the 365 days of the year	**−1.10**	−12.41	0.00	**−0.696**	−8.15	0.00
**Social behaviour characteristics**						
(14) Custom or habit since child	**−0.500**	−5.76	0.00	**−0.315**	−3.78	0.00
(15) My close family and friends also eat seabream/seabass	**−1.54**	−16.61	0.00	**−1.05**	−11.83	0.00
**Price**						
(16) Price	0.00	-fixed-	-fixed-	0.00	-fixed-	-fixed-
*Alternative specific constants (ASCs)*	Value		t-stat		*p*-value	
ASC1	**0.172**		5.44		0.00	
ASC2	**0.133**		4.39		0.00	
ASC3	**0.166**		5.74		0.00	
ASC4	0.00		-fixed-		-fixed-	
*Goodness of fit*						
*McFadden’s pseudo R* ^2^ *(ρ* ^2^ *)*	0.195					
*Adjusted McFadden’s pseudo R^2^ (Adjusted ρ^2^)*	0.193					
Final Log-likelihood	−14,039.592					
Number of observations	14,040					

Values in bold font indicate a significance level of 0.05, while those in italics indicate a significance level of 0.1.
